# Identifying the genes underlying quantitative traits: a rationale for the QTN programme

**DOI:** 10.1093/aobpla/plu004

**Published:** 2014-01-16

**Authors:** Young Wha Lee, Billie A. Gould, John R. Stinchcombe

**Affiliations:** 1 Department of Ecology & Evolutionary Biology, University of Toronto, Toronto, ON, CanadaM5S 3B2; 2 Centre for the Analysis of Genome Evolution and Function, University of Toronto, Toronto, ON, CanadaM5S 3B2

**Keywords:** Adaptation, ecological genomics, ecologically important traits, genetic variation, phenotypic evolution, population genomics, QTL, QTN, quantitative genetics, vertical integration.

## Abstract

The goal of identifying the genes or even nucleotides underlying quantitative and adaptive traits has been characterized as the ‘QTN programme’ and has recently come under severe criticism. Part of the reason for this criticism is that much of the QTN programme has asserted that finding the genes and nucleotides for adaptive and quantitative traits is a fundamental goal, without explaining *why* it is such a hallowed goal. Here we outline motivations for the QTN programme that offer general insight, regardless of whether QTNs are of large or small effect, and that aid our understanding of the mechanistic dynamics of adaptive evolution. We focus on five areas: (i) vertical integration of insight across different levels of biological organization, (ii) genetic parallelism and the role of pleiotropy in shaping evolutionary dynamics, (iii) understanding the forces maintaining genetic variation in populations, (iv) distinguishing between adaptation from standing variation and new mutation, and (v) the role of genomic architecture in facilitating adaptation. We argue that rather than abandoning the QTN programme, we should refocus our efforts on topics where molecular data will be the most effective for testing hypotheses about phenotypic evolution.

## Abstract (precis)

Identifying quantitative trait nucleotides (QTNs), the genetic polymorphisms linked to phenotypic variation, has become a goal for many plant ecologists and evolutionary biologists in recent years. But what is the true value of this potentially expensive and labor intensive programme of research? In this review we discuss the ways by which the QTN programme can offer unique insight into the ecology and evolution of adaptation in plants. We cite recent noteworthy examples of QTN work and provide recommendations for refocusing efforts to identify and study the genes underlying ecologically important traits.

## Introduction

A dominant goal of contemporary evolutionary genetics has been to describe, at the molecular level, the loci responsible for adaptations and complex phenotypes. While this goal first gained steam in non-model systems with the widespread adoption of quantitative trait locus (QTL) mapping in the late 1990s, the recent explosion of sequencing technologies is likely to only heighten interest in this goal. New sequencing technologies, analytical approaches and statistical methods now offer the prospect of detecting the nucleotide variants responsible for variation in quantitative traits—an agenda Rockman (2012) termed the QTN programme. It is easy to understand the excitement: adaptive phenotypes previously only understood statistically with variances, covariances and linear algebra might soon be understood as a collection of single-nucleotide polymorphisms (SNPs) whose inheritance, allele frequencies and evolutionary dynamics could be understood with the basic principles of population and transmission genetics.

Many, but not all, papers in contemporary ecological and evolutionary genetics take it for granted that describing the molecular basis of adaptations is a worthy goal—or simply assert that it is a fundamental goal—without providing an explanation for *why it is a worthwhile goal.* An important consideration for any QTN-based research is: What are the benefits of knowing the *specific* genes, genetic regions or nucleotides responsible for adaptive phenotypes above and beyond the generic knowledge that there must be *some unknown* genes, genetic regions or nucleotides that contribute? Here we aim to provide answers to this question, and as such a rationale for the QTN programme.

## Criticisms of the QTN Programme

Rockman (2012) strongly criticizes several aspects of the QTN programme, largely on the basis of logical flaws in how the results are interpreted. Most of the methods for detecting QTNs—QTL mapping, candidate gene association studies, genome-wide association studies (GWAS) and others—are strongly biased towards detecting nucleotide polymorphisms with a large effect on phenotype and overestimating their phenotypic consequences (i.e. the Beavis effect, see Slate 2013). As such, he argues that generality from QTN studies is likely to be elusive: the methods we use predispose us to find large-effect variants, giving a biased picture of their importance. Rockman argues persuasively that neither theory nor data support the notion that large-effect variants are the primary loci of adaptation, and that existing data suggest that many large-effect loci are likely to be qualitatively different at the molecular level than small-effect loci. Accordingly, Rockman argues that if the loci that we are able to find are a biased sample of the universe of QTNs, any conclusions we make about the evolutionary and genetic phenomena based on them are also likely to be biased.

Travisano and Shaw (2013) criticize the QTN programme on a more fundamental level. They argue that searching for QTNs holds little value in that such research focuses on patterns (e.g. genomic signatures of selection or statistical associations between sequence variants and phenotype) rather than the evolutionary process itself. Travisano and Shaw (2013) argue that intensive molecular studies ‘… have not altered fundamental understanding of the relationship between genotype and phenotype …’ nor yielded the ability to predict organismal evolution in response to selection. They argue that QTN searches are thus less worthy of investment than studies that are mechanistic in nature.

## What Now?

We suggest that the challenge posed by Rockman is one of properly interpreting the generality and inference space of QTN results. A dissecting scope and scanning electron microscope both provide enhanced magnification for studying biological features: one is useful for studying external morphology and the other for cellular and sub-cellular structures. The inability to describe cellular morphology with a dissecting scope does not imply that we should discard it, but rather only use it for tasks where it is appropriate. Are the existing QTNs—which Rockman argues are primarily (unrepresentative) large-effect loci—so well characterized that we can stop studying them? Below, we describe a rationale for why we should characterize the genetics, ecology and evolutionary dynamics of QTNs and what we can learn from them. While the smallest-effect, infinitesimal QTN might be beyond our reach right now (or perhaps, always), we seek to clarify what one can learn from any QTN and why they are worth pursuing.

We also endorse Travisano and Shaw's (2013) call for mechanistic studies of natural selection and adaptation that lead to changes in phenotypes. We see great value in this approach, and see no reason why it should not be paired with molecular genetic studies. The challenge, as we see it, is to design genetic studies to clarify aspects of the evolutionary process that are unavailable at the purely phenotypic level, and can enhance evolutionary interpretations.

## A Rationale for the QTN Programme

**Figure 1.**
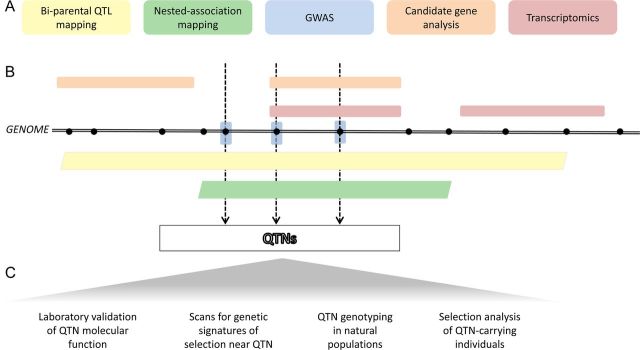
Schematic of common approaches to the QTN programme, with relative resolution of the methods. Major approaches used to identify QTN underlying ecologically important traits are listed in colour-coded boxes (A) (also described in Table 1). Each approach implicates genomic targets (i.e. single genetic polymorphisms, whole genes or genomic regions) that potentially underlie variation in the trait of interest. These targets are shown in relation to their position in the genome in (B), in the same colour as the corresponding method in (A). In (B), dots along the genome represent genetic variants (most often SNPs), which are used as markers in QTL, GWAS and candidate gene studies. Rectangles represent coding regions (genes), and parallelograms represent larger genomic regions. Variants in the genome that are implicated by multiple studies that use different methods are our best candidates for true QTN. These are highlighted with dotted arrows in (B). Following discovery of potential QTN, further analyses can then be undertaken (C) to verify the influence of each QTN on organismal phenotype and to explore their population genetic and ecological dynamics in natural settings.

Numerous reasons to seek QTNs exist, and here we explore in detail those that are most compelling to us. Throughout, we focus on work in natural (rather than agricultural or experimental) systems, seeking to highlight plant examples whenever possible. A summary of the strengths and weaknesses of the various approaches can be found in Table 1, and a schematic on the relative precision of alternative approaches in Fig. 1. We see at least five compelling reasons to seek QTNs: (i) understanding adaptive phenotypes across many levels of biological organization, from the nucleotide to the ecological and community context, a feature we refer to as *vertical integration* (M. A. Bell, pers. comm.); (ii) understanding genetic parallelism and the role of pleiotropy in constraining adaptation; (iii) understanding the maintenance of genetic variation; (iv) understanding the role of standing genetic variation in adaptation; and (v) understanding the role of genomic architecture in adaptation.

**Table 1.** Strengths and weaknesses of frequently used experimental methods in the QTN programme. LD, linkage disequilibrium.

Approach (potential resolution)	Method	Advantages	Disadvantages	Examples
Bi-parental crosses				
Fine mapping and positional cloning (QTN)	A QTL is introgressed into a homogeneous genetic background. Resulting lines segregate only within the QTL region (near isogenic lines). Recombinants are generated and tested for trait associations	• Very-small-effect variants can be resolved by progeny testing • Unhampered by the confounding factors and power issues arising in the population level association studies	• Time and labour intensive • Limited to measuring only two parental alleles per locus • Behaviour of QTLs as they are resolved into smaller regions is unpredictable—the association often disappears with increasing resolution or different genetic background • Linkage map required	Li *et al.* (2004)
Bulk segregant mapping (QTN)	A large recombinant bi-parental mapping population is created. Truncation selection is performed and the selected pools are sequenced and queried for shifts in allele frequency compared with the control	• Comparatively inexpensive as bulks can be sequenced in pools • Fast—QTNs are within reach with one generation of breeding in an F2 or backcross mapping population	• Large sample sizes mean that phenotyping is labour intensive • Limited to sampling two parental alleles • Resolution and power low for small-effect QTLs • QTN resolution requires a reference genome	Ehrenreich *et al.* (2010)
Nested association mapping (a few genes–a few cM)	Multiple parents are chosen and subject to a balanced crossing design that also seeks to maximize informative meioses. A high-resolution mapping population is created where all genomic segments have been shuffled relative to each other	• Allows population sampling while reducing the confounding effects of population structure • Rare variants accessible as their frequency is artificially increased • Can become a community resource	• Time and labour intensive to generate and maintain • Low general genomic resolution	McMullen *et al.* (2009)
Candidate gene association study (QTN)	A candidate gene is cloned starting with PCR primers based on a candidate gene sequence in another species. The gene is sequenced in natural population(s) using traditional Sanger or next-generation sequencing	• Fast—no need to generate mapping populations • Accessible for obligately outbreeding individuals • Information in literature on gene of interest	• Need prior knowledge of candidate genes • False positives due to unknown background factors or unaccounted for LD • Power low for small-effect and/or rare QTNs	Harjes *et al.* (2008)
Genome-wide association study (QTN)	Large population samples are either genotyped with a set of high-density markers or whole genomes are sequenced. Statistical models seek to associate genetic variants with trait variation while accounting for potentially confounding factors	• Large representative population samples • Accessible for obligately outbreeding individuals	• Expensive to sequence/genotype • False negatives in the process of accounting for multiple testing • False positives due to population or kinship structure • Power low for small-effect and/or rare QTNs • Requires a reference genome	Gudbjartsson *et al.* (2008) Lettre *et al.* (2008) Weedon *et al.* (2008) Atwell *et al.* (2010)
Transcriptomics (gene)	Expression levels of many/most genes in the transcriptome are measured using RNA sequencing, microarrays or other approaches. Expression variation for each transcript is associated with phenotype	• Less expensive and labour intensive than other approaches • Data specific to expressed portions of the genome • Tractable in non-model species	• Produces many significant targets • No estimate of effect size • Control for background genetic effects can be difficult	Ness *et al.* (2011) Liu *et al.* (2013)

### Vertical integration

Biologists of many stripes often repeat the famous Dobzhansky (1973) quote, ‘nothing in biology makes sense except in the light of evolution’, to refer to the unifying and explanatory power of evolution, across many levels of biological organization. And, it is indeed true that many features of biology, from the cellular and the molecular to broad-scale ecological patterns, can be understood from an evolutionary perspective. Yet, how many examples do we have where we understand the evolution of adaptation from the level of genetic variation at causal nucleotides, to heritability in natural populations, to the strength of contemporary selection, to how patterns of genetic variation and selection depend on geography and ecological context? We have a plethora of examples where a particular feature in this hierarchy of biological organization is understood, but not the rest of it. For example, in many systems we now have an exquisite understanding of genetic mechanisms and developmental processes underlying phenotypes (reviewed by Stern and Orgogozo 2008), but with far less knowledge of their ecological function and context. Likewise, many of the well-studied systems of evolutionary ecology have provided great detail on the ecological mechanisms of selection, as well as patterns of genetic variation and natural selection, in total ignorance of the molecular genetic basis of the traits or the developmental pathways producing them. Nearly 60 years after the discovery of DNA and almost 100 years after the evolutionary synthesis, it is remarkable that we have so few examples we can point to of complete vertical integration in our understanding of adaptive phenotypes.

In large part, what the QTN programme strives for is to generate these much-needed, empirically complete studies of ecological adaptation. Often this involves the identification of QTN for traits that have been studied at the phenotypic and ecological levels for decades, and has the potential to reveal unpredicted complexities of the evolutionary process. For example, recently the genetic basis of flower colour evolution was uncovered in two closely related wildflowers, *Phlox drummondii* and *Phlox cuspidatum*. In sympatric populations, an unusual dark red morph of *P. drummondii* occurs (both species are normally light pink), and work over the decades supported the hypothesis that the phenotype evolved as a result of classical reinforcement (selection for reduced inter-species hybridization) (Erbe and Turner 1962; Levin 1972, 1985).

In 2011 Hopkins and Rausher used a candidate gene approach to uncover QTN linked to flower colour in these species. They uncovered one mutation that is tightly associated with pigmentation (pink vs. red) and another for colour intensity (light vs. dark). They then returned to 39 natural populations, sequenced the QTN-containing loci and found nucleotide diversity patterns indicative of recent, strong natural selection at the red colour locus. No signature was found at the intensity locus and thus it seemed red flower colour had been verified as the target of selection (Hopkins *et al.* 2011). However, motivated by the identification of two separate QTN, they then generated plants carrying different combinations of the QTN for colour and intensity, introduced them back into natural settings and observed pollinator visitation to gauge selection pressures on the alleles. Unexpectedly, they found that pollinators imposed selection for intensity (darkness) but not colour itself (Hopkins and Rausher 2012). Vertical integration through QTN research in this system has revealed a more complex story than would otherwise have been appreciated as a result of either ecological or molecular studies in isolation. The reason red flower colour has evolved in conjunction with recent selection for dark colour intensity remains a mystery, perhaps to be solved by continued genetic work.

Similarly, vertical integration through a study of QTNs has expanded our understanding of adaptation and reproductive isolation in the common monkey flower (*Mimulus guttatus*) (Wright *et al.* 2013). In this species, populations growing on contaminated mine soils have evolved a high level of copper tolerance, and the trait co-occurs with tissue necrosis in hybrid offspring between on- and off-mine populations. The discovery that a single genomic region (QTL) was linked to variation in both traits made it seem probable that reproductive isolation between populations had been driven by genetic pleiotropy at one selected locus (Macnair and Christie 1983). However, continued genetic dissection of the QTN underlying the QTL showed that, despite known similarities between the genes that confer both heavy metal tolerance and hybrid necrosis in *Arabidopsis*, not one but two separate, physically linked loci control variation in the two traits. Genetic hitchhiking during a selective sweep at the copper tolerance locus captured alleles producing hybrid incompatibility between on- and off-mine populations. Only a detailed QTN approach was able to reveal the mechanistic connection between the evolution of adaptation and reproductive isolation.

The list of cases where ecologically important QTNs or genes have been identified and studied to some extent in natural settings continues to grow [e.g. *Boechera stricta* (Prasad *et al.* 2012), peppered moths (van't Hof *et al.* 2011; Cook and Saccheri 2013), various species of mice (Nachman *et al.* 2003; Hoekstra *et al.* 2006; Linnen *et al.* 2013), *Heliconius* butterflies (Baxter *et al.* 2010), stickleback fish (Colosimo *et al.* 2005) and many others (McKay and Stinchcombe 2008; Stern and Orgogozo 2008; Manceau *et al.* 2010; Martin and Orgogozo 2013)]. However, we argue on two fronts that discovery of QTN under selection is still a much-needed area of research. First, as Rockman noted, the phenotypes that have been linked to QTN thus far are heavily biased toward traits that are qualitative in nature (i.e. pink vs. red flower colour) and have a very simple genetic basis. Relatively few examples exist where selection on QTN for truly quantitative traits has been demonstrated. Effectively detecting QTN underlying complex traits is a challenging goal in ecological genetics, but one that is not out of reach. Genome-wide association studies in cultivated plants and *Arabidopsis* have already made great strides in this area through the use of large sample sizes and marker sets that are saturating across the genome (Atwell *et al.* 2010; Brachi *et al.* 2010). Second, rarely have any QTN been shown empirically to change in frequency through more than a few generations in natural populations, on time scales pertinent to the evolution of traits through ecological selection (but see Barrett and Schluter 2008), thus pairing the QTN approach with the direct observation of the evolutionary process advocated by Travisano and Shaw. Such a pairing requires analysis of preserved specimens, genetic sampling of QTNs in populations across years or other creative approaches. We predict that some of the most empirically complete examples of vertical integration yet to come will track traits and their underlying QTN through time in natural populations, providing unique examples of microevolution in action.

### Genetic parallelism and pleiotropy

QTN research has revealed captivating stories in the study of the underlying mechanisms of convergent phenotypic evolution across related organismal groups. Identifying genes and QTNs responsible for similar but evolutionarily independent phenotypic shifts in different taxa is the only way to directly test for the extension of convergence down to the nucleotide level. The degree of genetic parallelism has direct implications for the role of pleiotropy and evolutionary constraint in adaptation (Wake 1991; Hoekstra 2006; Des Marais and Rausher 2008, 2010; Samis *et al.* 2012). This is to say, if the same genes or mutations produce a common phenotype (genetic parallelism), it suggests that either (i) there are limited genetic mechanisms for producing the same trait due to developmental or structural constraints (Wake 1991), or (ii) repeated changes to specific genes or regions are favoured because changes to those regions have fewer deleterious pleiotropic side-effects relative to others (Stern and Orgogozo 2008). In contrast, if the same phenotype can be produced by many genetic mechanisms (genetic non-parallelism), it suggests genetic heterogeneity underlying adaptive phenotypes (Kelly 2006; Travisano and Shaw 2013). Theoretical work confirms the intuition that parallel evolution at the sequence level will be common when there are relatively few genetic mechanisms to produce the adaptive phenotype, and much more rare when there are many genetic routes to adaptation (Orr 2005).

There are still very few studies of the molecular basis of parallel phenotypic changes across taxa in wild plants, with one striking exception: the study of evolutionary transitions in flower colour. It has long been noted that within genera, two types of flower colour changes, from pigmented flowers to white flowers and from blue to red flowers, have occurred independently in many angiosperm clades. The molecular genetic basis of these changes has now been determined in at least 10 separate taxa (reviewed by Rausher 2008; Hopkins and Rausher 2011; Smith and Rausher 2011). Researchers have found that the evolution of parallel shifts in flower colour in the wild is often controlled by changes in the exact same loci in distant clades. Examples include comparisons of flower colour shifts between and within snapdragons, morning glories, monkey flowers, columbines and petunias, among others. While the nature of specific QTNs in these loci varies between species, evolution of flower colour is indeed parallel at the level of genes in many cases. Remarkable patterns of convergence in the evolution of morphological traits at the genetic level have also been recently discovered in cultivated plants (Ryan and Delhaize 2010; Lin *et al.* 2012), insects (Reed *et al.* 2011), fish (Colosimo *et al.* 2005; Hohenlohe *et al.* 2010; Colombo *et al.* 2013) and other vertebrates (reviewed by Hoekstra 2006). The observation of genetic parallelism in many cases, despite the known complexity of gene and protein networks that culminate in the realization of phenotypic traits, provides support for the role of pleiotropy in constraining evolution, or at least restricting it to certain loci.

### Maintenance of standing genetic variation

Even before mechanistically examining the alleles underlying adaptation, we might consider another question of fundamental importance in evolutionary biology: Why is there such an abundance of heritable variation in nature? Can the QTN programme help explain how it is maintained? The maintenance of standing genetic variation is one remaining major evolutionary question that lacks a significant body of empirical evidence to distinguish between alternative hypotheses (Lewontin 1974), despite a rich body of theory that provides genetic models of the underlying evolutionary dynamics. Such models can be classified into two categories that differ in the role of selection and its effect on variation. Under mutation–selection balance, the genetic variation observed in populations reflects an equilibrium between mutation that introduces deleterious variation and purifying selection that depletes it (reviewed by Johnson and Barton 2005). The variants maintained in the population in this case are expected to be composed mostly of rare, recessive alleles. In contrast, balancing selection hypotheses propose that negative and positive selection acting antagonistically on the same site for different components of the trait, or in different genetic or environmental contexts, result in the maintenance of both alleles. Balancing selection predicts polymorphisms at intermediate, sometimes stable frequencies. There are numerous scenarios that lead to this result: frequency-dependent selection (Hori 1993), overdominance induced by antagonistic pleiotropy (opposing effects on different fitness components; Rose 1982; Hedrick 1999) and variable selection on genotypes in heterogeneous spatial or temporal environments (Levene 1953). For reviews of the relevant theory, see Hedrick *et al.* (1976), Hedrick (1986) and Mitchell-Olds *et al.* (2007).

Before QTN studies, empirical work on the maintenance of standing variation fell naturally under the domain of quantitative genetics. While a few traits with a Mendelian genetic basis had been identified to be under balancing selection (e.g. cichlid handedness maintained by frequency-dependent selection; Hori 1993), most traits are quantitative. Estimates of heritability and artificial selection experiments have shown that abundant genetic variation exists for almost any given trait (Houle 1992; Merila and Sheldon 1999; Hansen *et al.* 2011), but there has been limited success in explaining the maintenance of that variation. Manipulative experiments have attempted to confirm the contributions of rare and/or recessive deleterious alleles (Charlesworth and Hughes 1999; Kelly and Willis 2001; Kelly 2003; Charlesworth *et al.* 2007) with equivocal results. For example, Kelly and Willis (2001) examined whether rare, recessive mutations accounted for genetic variation in flower size in the common monkey flower (Kelly and Willis 2001). In brief, inbred and outbred populations were subject to artificial selection, and changes in mean flower size and directional dominance (the direction of how the trait changes with inbreeding) were estimated. Rare recessive alleles will result in a larger change in the directional dominance of the trait relative to the change in the mean (Kelly 1999). They found that rare recessive alleles were not enough to explain the existing genetic variation in flower size, suggesting that intermediate-frequency alleles contribute to flower size variation, implicating balancing selection on flower size (and the alleles responsible for it). These results suggest a more complicated interplay of selective forces than previous experiments that showed the population carried a large amount of partially recessive genetic load (Willis 1999*a*, *b*, *c*), suggesting mutation–selection balance. While tantalizing, these methods that rely on the estimation of net inbreeding load or variance components do not disentangle the contributions of mutation, negative selection, positive selection and balancing selection acting on many different loci that affect traits, and will not result in a comprehensive understanding of the balance of forces in the maintenance of variation. In contrast, identifying and directly studying selection on small- and large-effect QTN for flower size and other traits in the field has this potential.

By identifying the causal sites underlying standing genetic variation, the QTN programme provides the opportunity to examine how selection acts on standing variation (and by inference, on variation in the associated traits), thereby connecting theory, quantitative genetics and population genetics. For instance, consider the prediction that QTNs maintained by mutation–selection balance will be rare, partially recessive and deleterious. An empirically testable hypothesis is that individuals with extreme phenotypes carry a greater number of mutations affecting that trait, a hypothesis that is now supported by QTN studies of human disease (Ji *et al.* 2008; Diogo *et al.* 2013). In these studies, candidate genes identified through GWAS were surveyed through deep population resequencing to identify rare putatively functional mutations. When ‘case’ and ‘control’ groups of individuals were compared, the case population was enriched in rare mutations. While the related notion that individuals with extreme phenotypes carry more deleterious mutations figures prominently in quantitative genetic models of apparent stabilizing selection (McGuigan *et al.* 2011), as of yet we know of no explicit tests of these predictions for quantitative traits thought to be under selection in nature. Comparisons of the number of functional mutations (genome wide or in the subset of genes in the molecular pathway determining the trait) carried by individuals with extreme phenotypes and those in the centre of the phenotypic distribution could be accomplished without bias due to QTN effect size. If a relationship was found, the implication is that at least some genetic variance is due to neutral or deleterious variants with pleiotropic effects on the focal trait.

The QTN programme also has the potential to determine the importance of the maintenance of genetic variation due to balancing selection, as knowledge of the causal QTNs enables the testing of specific theoretical models. Direct evidence includes a demonstration of heterozygote advantage at the selected locus or the absence of a genotype that has maximum fitness in all environments and/or genetic backgrounds. In the special case of frequency-dependent selection, it requires demonstration that the minor allele is always advantageous. Empirically detecting this is a challenging endeavour, and recent years have seen progress on detecting fitness trade-offs at the scale of QTL (reviewed in Lowry 2012), including within-population QTLs (Mojica *et al.* 2012), though fitness effects attributed to QTLs may be confounded by linkage. The best examples are the culmination of years of fieldwork (Johnston *et al.* 2013) or benefit from a strong history of molecular genetics that implicate candidate genes for further experiments in the field (Tian *et al.* 2003; Todesco *et al.* 2010). These studies are classic examples of ‘vertical integration’.

While such cases of ‘vertical integration’ are of great value, the QTN programme also offers an alternative route to testing the general importance of balancing selection for maintenance of variation in quantitative traits. Population genomic scans for the molecular imprint of balancing selection (see Charlesworth 2006) combined with a systematic search for QTNs affecting a variety of traits thought to be components of fitness variation in nature are an additional method of evaluating the role of balancing selection. Diagnostic molecular population genetic signatures of balancing selection include increased diversity in sites linked to QTN under long-term balancing selection, the existence of shared segregating haplotypes across related species or a common haplotype harbouring little variation for an allele that recently increased in frequency (see Charlesworth 2006 for a review). Dense genotype data have facilitated genome scans in human populations (Sabeti *et al.* 2002; Andres *et al.* 2009; Leffler *et al.* 2013), but without the overlap of candidate genes it is unknown what traits are affected by the regions under selection (Stinchcombe and Hoekstra 2008). While differentiating the signal of selection from neutral variation in these data sets is a challenge, the ability to combine a catalogue of QTNs where we have estimates of trait heritability with molecular population genetic tests for balancing selection would provide exciting advances in our understanding of the maintenance of standing genetic variation in the wild.

### Role of standing genetic variation in adaptation

Evolutionary responses to selection will differ greatly depending on whether the substrate of adaptation comes from either new mutations or standing variation. In the first case, adaptive divergence would be dependent on the rate of occurrence of new beneficial mutations. However, if the standing variation provides the substrate of adaptation, the evolutionary dynamics are determined by the factors that convert the standing variation into beneficial alleles, or change the magnitude of the selection coefficient. Consequently, a key question—regardless of the initial allele frequencies—is what changes to the ecological environment or selective regime make alleles sufficiently beneficial that they increase in frequency and fix. If the alleles that fix had previously been maintained by balancing selection, evolutionary responses will be influenced, at least in part, by how standing variation is maintained.

An elegant example of how to combine QTN and molecular population genetic scans for selection was provided by a recent study using European-wide and local *Arabidopsis* accessions (Fournier-Level *et al.* 2011), and is an example of the way forward to a more general assessment of the contribution of standing variation to adaptation. Fixation events in the course of adaptation are expected to leave a characteristic molecular footprint on linked sites, where adaptation from new mutation is accompanied by the signature of a ‘hard sweep’, i.e. a steep decline in nucleotide diversity around the fixed site characterized by an excess of rare alleles close to the selected site. Fixation events from the standing variation are accompanied by the signature of a ‘soft sweep’, where a lesser decline in diversity around the fixed site is also associated with more intermediate-frequency alleles due to selection acting on multiple haplotypes containing the causal QTN (Przeworski *et al.* 2005). Fournier-Level *et al.* grew a large collection of accessions in four geographically distinct regions that spanned the climatic range limits in Europe, and QTNs for fertility and viability were identified in each region. Interestingly, the authors found that the alleles increasing fitness generally did not overlap between regions. Fournier-Level *et al.* also found that some of the beneficial alleles were naturally more abundant locally than the geographic distribution of a set of control SNPs. These results are consistent with local adaptation; however, there was limited evidence for the signal of recent ‘hard’ selective sweeps from new mutations around those sites in the local samples. These results imply that local adaptation was due to the differences between regions in the standing variation, and that evolutionary dynamics within and among different populations were not limited by the occurrence of new mutations.

The power to detect QTNs is determined by how much phenotypic variance is explained by the QTN, which is in turn determined by the effect size and allele frequency of that QTN. Thus the identification of QTNs is unavoidably limited by genetic architecture. The empirical results to date suggest that the genetic architecture of plant traits often includes major-effect alleles, with the caveat that plant GWAS to date have focused on crop species that underwent population bottlenecks and strong artificial selection, landraces with histories of introgression, and selfers such as *Arabidopsis*. Plant GWAS to date typically have smaller sample size (<1000) and yet successfully identify handfuls of loci that explain significant trait variance and overlap previously identified candidate genes (Aranzana *et al.* 2005; Chan *et al.* 2011; Famoso *et al.* 2011; Filiault and Maloof 2011; Huang *et al.* 2011; Morris *et al.* 2013; Rosas *et al.* 2013; Stanton-Geddes *et al.* 2013). For example, a GWAS of rice landraces (*n* = 517) identified QTNs for 14 traits that explained on average 36 % of trait variation (Huang *et al.* 2010). Similarly, a GWAS for 107 traits in *Arabidopsis* (*n* = 96 or 192 individuals depending on the trait) identified numerous common major-effect alleles underlying trait variation (Atwell *et al.* 2010; also see Bergelson and Roux 2010 for an overview). While most of these identified associations remain to be substantiated by further experiments (for example, in crossing designs or functional analysis), these data to date suggest that it is possible to identify QTNs in plants that explain a significant proportion of the phenotypic variance in natural populations. These results, however, do not obviate the contribution of small-effect loci underlying heritability in plants; for example, a 70-generation selection experiment for higher and lower oil content in maize showed that the sustained response was due to many small-effect loci (Laurie *et al.* 2004).

While the utility of GWAS for identifying genetic sources of trait variation has been demonstrated, we suggest the QTN programme expand to include work that focuses on sampling within (rather than between) populations, to identify the QTNs that are of the most evolutionary and ecological significance. The QTNs that we are able to identify are those that explain a significant portion of phenotypic variance for the trait. The contribution of a given biallelic locus to the additive genetic variance in a trait is given by 2*pqα*^2^, where *p* and *q* are the frequencies of alternative alleles, and *α* is the average effect (Lynch and Walsh 1998). Consequently, common alleles will be the primary drivers of the initial selection response and will be of the most evolutionary interest, at least until rare alleles that also affect fitness increase in frequency. In addition, large-effect alleles—which might be in the minority of all alleles affecting a trait, or that are ultimately involved in the selection response—can make significant contributions to the genetic variance and evolutionary response, simply because of their effect size. To date most GWAS in plants have been composed of global samples that seek to maximize genetic and phenotypic variance, and identify causal associations while controlling for broad-scale population structure. An implicit assumption in these experimental designs is that causal alleles are widespread across many populations. However for quantitative traits, the same phenotypes in different populations may have a different genetic basis. Such global samples will cause even common alleles in local populations to be rare in the global sample if they are of limited geographic distribution, as would be expected if local adaptation is common. Thus except for globally common alleles, the frequency of any given allele is lowered, and the power to detect locally significant QTNs may be diluted. The QTNs identified in such global samples will also not necessarily be the ones underlying local selection responses. Even with power limitations, if the QTN programme includes studies aimed at understanding the selection response, and not simply finding genes, even those few QTNs we identify will lead to insight into the evolutionary process. As within-population sampling becomes more common, one area of future development will likely be methods for controlling for close kinship between individuals (e.g. Manichaikul *et al.* 2010).

### Understanding the role of genomic architecture in adaptation

The QTN programme can also clarify the role of aspects of genomic architecture—chromosomal inversions, translocations, ‘supergenes’ in areas of suppressed or restricted recombination—in adaptation (Kirkpatrick and Barton 2006; Scoville *et al.* 2009; Yeaman 2013). In particular, the potential role of supergenes in adaptive phenotypes has been suspected since the dawn of plant genetics, and a robust set of theoretical predictions and population genetics has developed in these areas. Moreover, turning these suspicions into testable hypotheses, and evaluating our theoretical predictions, is something that can only be achieved with a molecular approach.

Early work on the genetics of heterostyly is illustrative (e.g. Bateson and Gregory 1905; Gregory *et al.* 1923; De Winton and Haldane 1933, 1935; Mather 1950). Heterostylous plants are polymorphic for the reciprocal arrangement of anthers and stigmas along with pollen size and other floral characteristics, with successful fertilization usually only possible between anthers and stigmas at the same level (long styles and pollen from long-level anthers; short styles and pollen from short-level anthers; see Barrett 1992 and Barrett and Shore 2008 for more details). Current evidence suggests that heterostyly functions to increase the proficiency of cross-pollination, promoting disassortative mating between the floral morphs (Kohn and Barrett 1992; Lloyd and Webb 1992). Genetic crossing designs strongly suggest one (distyly) or two (tristyly) diallelic loci governing the style–stamen polymorphisms. The complex morphological and physiological components of the heterostyly syndrome, along with observations that recombinants are rare, have suggested supergene control involving a co-adapted linkage group (Lewis and Jones 1992).

There are numerous challenging evolutionary questions regarding the evolution of heterostylous pollination systems. How did these intricate, seemingly co-adapted systems evolve? Are initially unlinked loci captured by chromosomal inversions or translocations, or must the loci already be loosely linked? How do selection and recombination interact in creating and breaking up these supergene complexes? How much pleiotropy is required for diverse features of floral morphology to be inherited together? These questions have received intense investigation from the theoretical population genetics perspective (Charlesworth and Charlesworth 1976; Charlesworth 1979; B. Charlesworth and D. Charlesworth 1979; D. Charlesworth and B. Charlesworth 1979). The construction of linkage maps and estimation of recombination rates and signatures of selection in genomic regions affecting floral phenotypes would help test the ‘supergene’ hypothesis. Consequently, the QTN programme—or at a minimum, molecular markers, linkage maps and molecular population genetic inference—can clarify the genetic basis and evolutionary forces responsible for polymorphisms that directly influence plant mating and fitness. For example, the generation of linkage maps can reveal whether marker order near style length QTL regions is reversed in different populations, suggesting chromosomal inversions. If inversions do not explain transmission patterns, molecular population genetic data on patterns of polymorphism, linkage disequilibrium (LD) and recombination can reveal whether loci influencing heterostyly are in regions of suppressed recombination. Similarly, population genetic analyses can test whether these regions harbour elevated divergence and LD indicating the long-term maintenance of a supergene cluster. We see these research questions as ones in which classical approaches and theory have led to clear, testable predictions that can be answered by the QTN programme, and where the conclusions are robust to issues of effect size and bias (since the predictions do not hinge on detection of small-effect QTNs). To date this approach has been successfully applied to other systems where supergenes have been invoked (mimetic butterflies: Joron *et al.* 2006, 2011; Counterman *et al.* 2010; shell colour and banding in snails: Richards *et al.* 2013).

Another area where the QTN (or QTL) programme is necessary for evaluating the role of genomic architecture in adaptation is in studies on the role of chromosomal inversions in local adaptation. Theoretical work by Kirkpatrick and Barton (2006) showed that chromosomal inversions could ‘capture’ locally advantageous haplotypes and spread quite rapidly. In this manner, several genes or loci that lead to local adaptation would be preserved together, largely because of the suppressed recombination that often accompanies inversions. These loci are not required to interact epistatically, genetic drift is not required, and there is no trough in the adaptive landscape to traverse (Kirkpatrick and Barton 2006). Kirkpatrick and Barton's theory was a significant advance in our expectations for the evolutionary dynamics of inversions, which previously assumed that they would be generally deleterious due to meiotic imbalance or disrupted genes at the breakpoints (Kirkpatrick 2010). For an overview of the role of chromosomal inversions in diverse topics in evolutionary biology, see Kirkpatrick (2010) and Kirkpatrick and Kern (2012). The discovery of a positively selected chromosomal inversion in humans (Stefansson *et al.* 2005) was in fact an early basic science ‘spin-off’ discovery facilitated by human GWAS studies (Visscher *et al.* 2012).

One of the best examples of a locally adaptive inversion is in the yellow monkey flower, *M. guttatus*, which inhabits a broad geographic range in western North America. Inland ecotypes tend to be annual, while coastal ecotypes are perennial, and the two types also differ significantly in flowering time. The divergent flowering time between inland and coastal ecotypes generates strong natural selection when the opposite type is experimentally transplanted: late-flowering, perennial coastal plants fail to reproduce before droughts in inland habitats, while early-flowering, annual inland plants fail to capitalize on the extended growing season provided by the coastal climate. Initial observations (Hall *et al.* 2010) suggested an area of suppressed recombination in a recombinant inbred line mapping population made from an inland × coastal cross. Subsequent work by Lowry and Willis showed that marker order was reversed between inland × inland and coastal × coastal crosses, and that recombination (as reflected by genetic map distances) was suppressed in inter-ecotype crosses, but not within ecotypes. Finally, ambitious ecological genetic field experiments showed that the inversion influenced flowering time, morphological characteristics and fitness in the field (Lowry and Willis 2010). The design used by Lowry and Willis (2010) allowed them to estimate the relative contribution of the inversion to reproductive isolation between the two ecotypes. In this case, a detailed QTN- or QTL-based approach again revealed fundamental insights into the evolutionary process—local adaptation and reproductive isolation due to chromosomal inversions—that were unattainable without the aid of molecular tools.

## Conclusion

The continuing advance of next-generation sequencing technology and its subsequent drop in price has opened the flood gates for research into the genes underlying trait variation in nature. Concomitant with this rush, some of the deeper reasons why we are interested in this information sometimes get lost, and often the caveats and limitations of QTN work are brushed aside in the wake of excitement over the seemingly endless possibilities. We need to reconvene, refocus and then redouble our efforts to gain insight into the evolutionary process, using genetic data where it can be the most effective and allow greatest testing of evolutionary hypotheses. Refocusing and planning efforts in the QTN programme must take into careful consideration not only these broader contexts, but the experimental resolution of available methods, their inference space and generality (Rockman 2012), and whether the outcome will generate a starting point for the essential mechanistic work advocated by Travisano and Shaw (2012). As evolutionists we see not the end to the utility of QTN work but an exciting future in which we are able to directly address how ecological and genetic factors interact over evolutionary time to generate and maintain phenotypic diversity.

## Sources of Funding

This work was supported by NSERC Canada and Genome Canada.

## Contributions by the Authors

All authors conceived the ideas, wrote the paper, edited manuscript drafts and responded to reviewer comments.

## Conflicts of Interest Statement

None declared.
